# Cooling Between Exercise Bouts and Post-exercise With the Fan Cooling Jacket on Thermal Strain in Hot-Humid Environments

**DOI:** 10.3389/fphys.2021.640400

**Published:** 2021-02-16

**Authors:** Hidenori Otani, Makoto Fukuda, Takehiro Tagawa

**Affiliations:** ^1^Faculty of Health Care Sciences, Himeji Dokkyo University, Himeji, Japan; ^2^ASICS Institute of Sport Science, Kobe, Japan

**Keywords:** body temperature, cooling garment, fan cooling, heat stress, exercise

## Abstract

This study investigated the effects of cooling between exercise bouts and post-exercise with a commercially available fan cooling jacket on thermal and perceptual responses during and following exercise in hot-humid environments. Ten male athletes completed two 30 min cycling bouts at a constant workload (1.4 watts⋅kg^–1^ of body mass) with a 5 min recovery period in between. Exercise was followed by a 10 min recovery period. In an environmental chamber (33°C, 65% relative humidity), participants performed two trials with (FCJ) or without (CON) the fan cooling jacket on a T-shirt during the 5 min inter-exercise and 10 min post-exercise recovery periods. Mean, chest and upper arm skin temperatures, and thermal sensation and comfort were lower in FCJ than CON trial during and following exercise (*P* < 0.05). Thigh and calf skin temperatures, infrared tympanic temperature and heart rate were lower in FCJ than CON trial during the experimental trials (*P* < 0.05). The rates of fall in mean, chest and upper arm skin temperatures, infrared tympanic temperature and thermal sensation and comfort were faster in FCJ than CON trial during both recovery periods (*P* < 0.05). There were faster rates of fall in thigh and calf skin temperatures and heart rate in FCJ than CON trial during the post-exercise recovery period (*P* < 0.05). No difference was observed between trials in the rating of perceived exertion (*P* > 0.05). This study indicates that cooling between exercise bouts and post-exercise with the fan cooling jacket would effectively mitigate thermal strain and perception/discomfort during and following exercise in hot-humid environments. This garment would reduce whole-body skin temperature quickly while promoting falls in lower-body as well as upper-body skin temperatures.

## Introduction

Human body temperature regulation is challenged during exercise (Maughan, 2010) and work (Kjellstrom et al., 2016) in heat stress environments. It has been predicted that around 30% of the world’s population is nearly exposed to climate conditions that exceed human thermoregulatory capacity for at least 20 days a year (Mora et al., 2017). That probably leads to a significant increase in morbidity and mortality (Mora et al., 2017). Cooling interventions are known to be the gold-standard method to reduce the risk of thermal strain associated with increases in body temperature during exercise-/work-heat stress. In sporting settings, pre-exercise cooling and during exercise cooling (per-cooling) in the heat have been recognized to mitigate thermal strain and enhance endurance performance (Tyler et al., 2015; Stevens et al., 2017; Douzi et al., 2019). Post-exercise cooling in the heat has been known to reduce the increased thermal strain quickly (McDermott et al., 2009; Bongers et al., 2017).

Intermittent sports have mandated breaks between exercise bouts as the 15 min half-time break in soccer/football and the 90 s changeover and 120 s set breaks in tennis. During these breaks, internal cooling with ice slurry ingestion (Naito et al., 2018), external cooling with an ice-vest (Chaen et al., 2019), and combined both with chilled water ingestion and iced towels (Chalmers et al., 2019) have been shown to reduce thermal strain during subsequent exercise in the heat. These studies reported that cooling between exercise bouts lowered core temperature (T_*core*_) (Naito et al., 2018; Chalmers et al., 2019), mean skin temperature (T_*sk*_) (Chaen et al., 2019) and heart rate (HR) (Naito et al., 2018; Chaen et al., 2019; Chalmers et al., 2019) during exercise compared to without cooling. With electric fan cooling between exercise bouts, fan use with skin wetting by externally applied water to the skin surface at inter-exercise has been demonstrated to attenuate thermal strain during exercise in uncompensable heat stress (e.g., the body is unable to maintain a thermal steady state) (Mitchell et al., 2001; Schranner et al., 2017; Lynch et al., 2018); to our knowledge, that has not yet been tested in compensable heat stress. Lynch et al. (2018) compared the effects of fan use with or without skin wetting on thermoregulatory responses during the 90 s changeover and 120 s set breaks in simulated tennis match-play in hot-dry conditions (45°C, 9% relative humidity). The authors found that fan use with skin wetting could be an effective strategy to mitigate increases in T_*core*_, T_*sk*_, and HR, but not without skin wetting. This would be associated with a greater evaporative heat loss from the skin with increasing airflow (Havenith et al., 1999). In support of this, whole-body fanning during exercise has been reported to mitigate increases in T_*core*_, T_*sk*_, and HR associated with enhancing evaporative heat loss with increasing airflow in compensable heat stress (Saunders et al., 2005; Otani et al., 2018b). Moreover, whole-body fanning at post-exercise in compensable heat stress has been shown to be the most effective method to reduce the increased T_*core*_ and T_*sk*_ accompanied by the greatest evaporative heat loss compared to hand cooling and the use of several cooling garments (Barwood et al., 2009). Meanwhile, frontal body fanning in seated posture has been demonstrated to expand the range of physiologically compensable ambient conditions delay elevations in T_*core*_ and HR at 36°C with ∼80% relative humidity and 42°C with ∼50% relative humidity (Ravanelli et al., 2015). This observation indicates that fanning the frontal body surface would make uncompensable heat stress conditions compensable during sitting in front of a fan.

**FIGURE 1:**
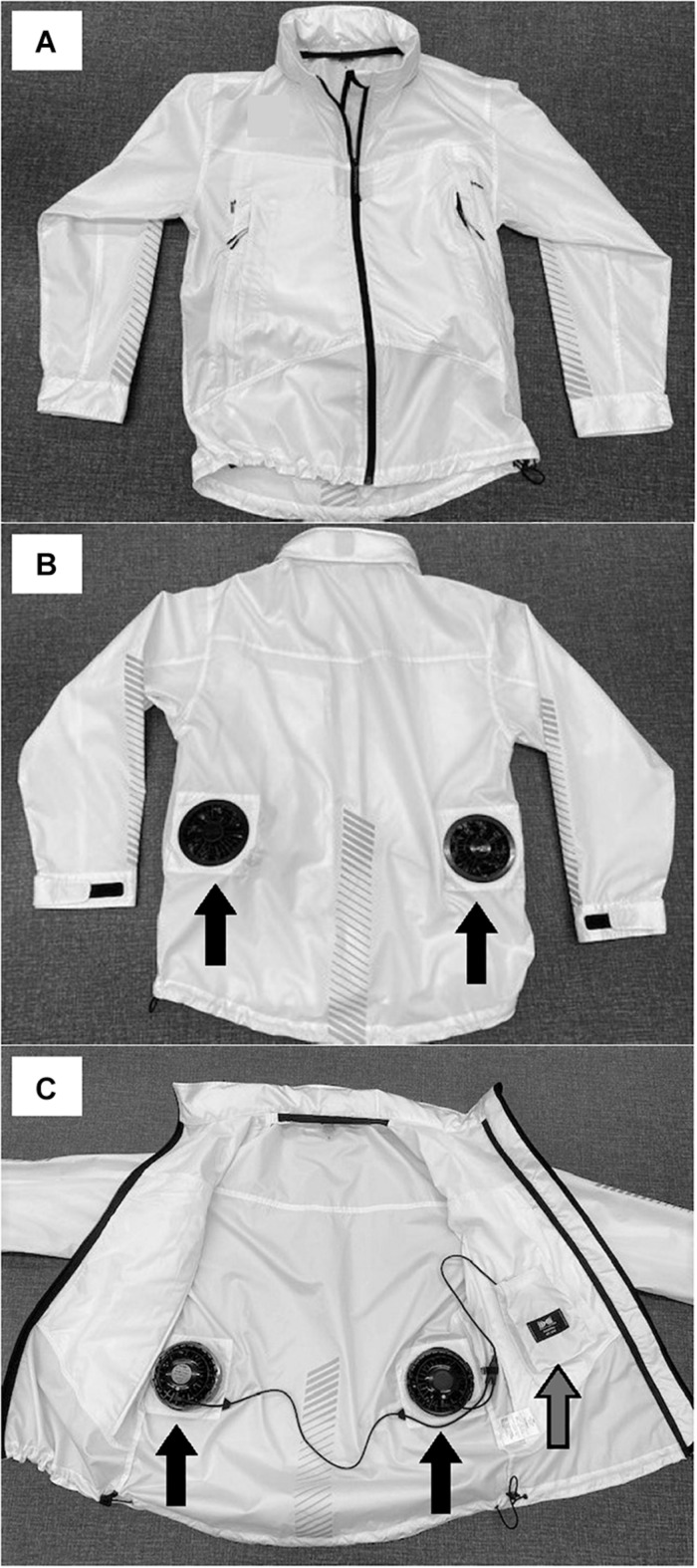
The fan cooling jacket. **(A)** anterior aspect. **(B)** posterior aspect. **(C)** anterior opening aspect. The black arrow indicates the fan **(B,C)**. The silver arrow indicates a battery box **(C)**.

Since a long time ago, the effects of auxiliary air cooling with the ambient air ventilated garment on thermal strain during light-intensity exercise in the heat were investigated in occupational and military settings (e.g., Christie et al., 1957; Shapiro et al., 1982). These studies showed that the ambient air ventilated garment use resulted in a smaller physiological strain with increasing the rate of ventilating air in both compensable and uncompensable heat stresses (Christie et al., 1957) and contributed to a lower T_*core*_ in comparison to no cooling in uncompensable heat stress (Shapiro et al., 1982). Recently, a newly developed fan cooling jacket/vest is commonly used in Japanese manual workers to mitigate the risks of thermal strain and exertional heat-related illness during work in the heat, especially in outdoors. This type of garment can induce fan cooling via the process of circulating airflow underneath clothing by two small fans on the lower back (Figure 1). To date, in simulating occupational heat stress, Wang et al. (2019) showed that the fan cooling garment use during walking in compensable heat stress was effective to attenuate increases in T_*core*_, T_*sk*_, and HR compared to no cooling. This fan cooling jacket will be useful cooling devices to reduce these risks in the heat in sports settings. Given that the observations of previous studies as described above (Barwood et al., 2009; Lynch et al., 2018), the use of the fan cooling jacket may also be more useful with skin wetting by sweating at inter- and post-exercise rather than pre-exercise. However, the effects of the fan cooling jacket use at inter- and post-exercise on thermal strain during and following exercise-heat stress have never been examined.

Therefore, the current study aimed to investigate the effects of cooling between exercise bouts and post-exercise with a commercially available fan cooling jacket on thermal and perceptual responses during and following exercise in compensable hot-humid environments. We hypothesized that the use of this jacket at inter- and post-exercise would reduce thermal strain and perception/discomfort during and following exercise compared with no use.

## Materials and Methods

### Participants

Ten healthy male athletes (eight tennis and one soccer players and one long distance runner) were recruited for the study (mean ± standard deviation [SD]; age 25 ± 6 y, height 172 ± 6 cm, body mass 67 ± 7 kg, BMI 22.6 ± 2.4 kg⋅m^–2^). Participants trained 5-7 days per week and were familiar with cycling exercise. All participants applied to the performance level 3 or 4 of the participant group classification by De Pauw et al. (2013). Participants gave their written informed consent. The ASICS Ethical Advisory Committee approved all experimental procedures (REF: 19-0024) and confirmed to the Declaration of Helsinki.

### Experimental Design

**FIGURE 2:**
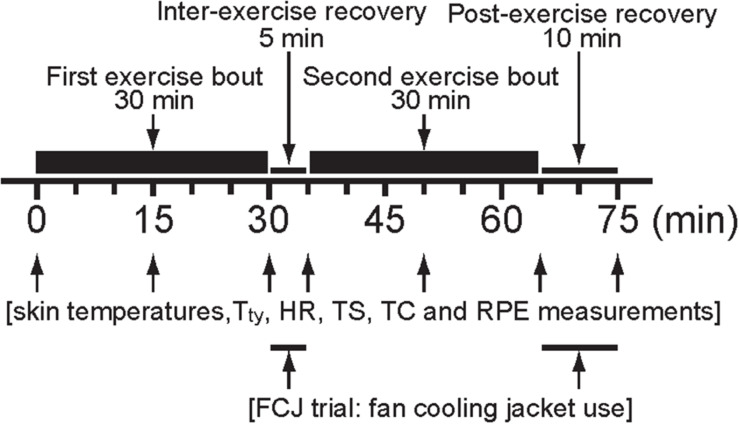
Schematic representation of the experimental protocol. T_*ty*_, infrared tympanic temperature; HR, heart rate; TS, thermal sensation; TC, thermal comfort; RPE, rating of perceived exertion.

All experimental trials were conducted between 10:00 to 15:00 h to minimize the time-of-day effects on thermoregulatory responses during exercise-heat stress (Otani et al., 2018a) and from mid-June to early-October 2020. Experimental trials were undertaken in an environmental chamber maintained at 33°C ambient temperature, 65% relative humidity, <0.3 m⋅s^–1^ air velocity, and 29°C wet-bulb globe temperature. The familiarization trial was undertaken without the fan cooling jacket to ensure the participants were accustomed to the procedures employed during the investigation. This trial was identical to the experimental trials in all respects. The experimental trial consisted of the first 30 min exercise (the first exercise bout: 0-30 min), 5 min inter-exercise recovery (the inter-exercise recovery: 30-35 min) and second 30 min exercise (the second exercise bout: 35-65 min) and 10 min post-exercise recovery (the post-exercise recovery: 65-75 min) periods (Figure 2). Participants cycled for the first and second exercise bouts using a cycle ergometer (Aerobike, Combi 75XL, Tokyo, Japan) at a constant workload of 1.4 watts per kg of body mass with a preferred pedal cadence between 60 and 70 rev⋅min^–1^. Participants performed two experimental trials with (FCJ), or without (CON) the fan cooling jacket (Figure 1: for more details, see Cooling garment section) on a T-shirt during the inter-exercise and post-exercise recovery. During these recovery periods, participants sat up straight on a stool with their hands on their knees. Participants were dressed in the same tennis ensemble (short-sleeve, crew neck T-shirt, men’s brief, shorts, crew-length socks and athletic shoes) in both trials. This tennis ensemble was 1.602 kg of total weight and 0.074 W⋅(m^2^⋅°C)^–1^, or 0.48 clo of the intrinsic clothing insulation (Zuo and McCullough, 2004). Participants completed two experimental trials in a counterbalanced order. Experimental trials were separated by at least 7 days. No exercise or alcohol consumption was permitted in the 24 h before the trials.

### Experimental Protocol

Participants entered the laboratory after a 3 h fast in both trials, other than the ingestion of plain water. Upon arrival, participants first emptied their bladder, and nude body mass was measured to the nearest 10 g (AD6205B, A&D Co., Ltd., Tokyo, Japan). Surface skin temperature thermistor probes (ITP082-25, Nikkiso-Therm Co., Ltd., Musashino, Tokyo, Japan) were attached to four sites at the chest (T_*che*_), upper arm (T_*upp*_), thigh (T_*thi*_), and calf (T_*cal*_). A heart rate (HR) telemetry band (Polar A370, Polar Electro, Kempele, Finland) was positioned around the wrist.

Participants then entered the environmental chamber and rested on a cycle ergometer in a seated position for 10 min. After this period, skin temperatures were recorded using a thermometer (N543R, Nikkiso-Therm Co., Ltd., Musashino, Tokyo, Japan), and HR was recorded. The current study used an infrared tympanic temperature (T_*ty*_) to estimate T_*core*_. T_*ty*_ was measured using an infrared tympanic thermometer (Genius^TM^ 2, Covidien, Mansfield, MA, United States), and two consecutive readings were obtained in each T_*ty*_ measurement. All T_*ty*_ measurements were taken by a single operator, using the recommended technique (Otani et al., 2005, 2020). Thermal sensation (TS) with a 9-point scale (ISO, 1995) and thermal comfort (TC) with a 4-point scale (Zhang et al., 2007) were measured after the rest period. After that, participants performed a 2 min self-paced warm-up at light intensity and commenced the first exercise bout. Rating of perceived exertion (RPE) was assessed during the trials using the 6-20 RPE scale (Borg, 1982) to determine the perception of effort. Skin temperatures, T_*ty*_ and HR were recorded, and TS, TC and RPE were assessed at 15, 30, 35, 50, 65, 70, and 75 min during the trials. Upon cessation of the first exercise bout, participants stood up from a cycle ergometer and took one/two steps to sit on a stool to initiate the inter-exercise recovery. After this period, participants returned to a cycle ergometer to initiate the second exercise bout. Upon cessation of the second exercise bout, participants again moved to sit on a stool to initiate the post-exercise recovery. After this period, participants exited the environmental chamber, removed the probes, toweled dry and re-measured nude body mass. Participants could consume water *ad libitum* during the trials, and it was maintained at about 30°C.

### Cooling Garment

Cooling was achieved with a commercially available fan cooling jacket (Air condition wear, ASICS, Kobe, Japan) (Figure 1). This jacket was the long-sleeve windbreaker/jacket and made from 100% polyester fiber. The garment induced fan cooling via the process of circulating airflow underneath clothing by two small fans (8 cm blade diameter) on the lower back. Two small fans could take in outside air and exhaust air from a cuff and neck. Two small fans accepted a battery box (6.5 cm × 8.5 cm: 4 AA nickel-metal hydride batteries) and were 0.44 kg of total weight (0.22 kg of each weight). The airflow rate of fans is 17.8 L⋅s^–1^ or 37.7 CFM. Airflow velocity underneath clothing is 5.4, 6.2, 3.0, and 7.6 m⋅s^–1^ at the palm side cuff, backside cuff, in front of the neck and behind the neck, respectively; this was measured using a micro anemometer (GeY-40DA, Tohnic Co., Ltd., Chigasaki, Japan). The fan cooling jacket consists of 0.82 kg of total weight. The intrinsic clothing insulation value for this jacket was 0.036 W⋅(m^2^⋅°C)^–1^ or 0.23 clo and was determined using a thermal manikin (Thermal manikin, Kyoto Electronics Manufacturing Co., Ltd., Tokyo, Japan) with a uniform skin temperature of 33°C and environmental temperature of 21°C and 50% relative humidity.

### Calculations

T_*sk*_ was calculated using the following equation (Ramanathan, 1964):

Tsk= 0.3⋅Tche+ 0.3⋅Tupp+ 0.2⋅Tthi+ 0.2⋅Tcal[C∘].

Total sweat loss was estimated using the following equation:

Totalsweatloss=bodymassloss+thevolumeofwateringested[kg].

It is recognized that this will introduce a small error as no account is taken of substrate loss (Maughan et al., 2007) and respiratory water loss, but this will be relatively small and rather constant between trials.

### Statistical Analyses

An *a priori* sample-size calculation was performed (G^∗^Power3.1.9.6; Dusseldolf, Germany) using data from previous investigations undertaken employing the similar experimental model (Otani et al., 2005, 2018a,b, 2020). This indicated that we would need ≥8 participants per group to find statistical significance with an effect size of 0.4, a power of 0.9 and alpha set to 0.05.

The first exercise bout of each trial was identical and classified as a control period. Therefore, the 30 min mark was treated as a baseline. Pre-exercise and the first exercise bout data were also analyzed and reported. The rates of fall in all variables were analyzed during the inter-exercise and post-exercise recovery.

Data are presented as mean ± SD. The significance level was set at *P* < 0.05. The normality of the data and the homogeneity of variance between trials were tested using Shapiro-Wilk’s test and Levene’s test, respectively. Non-parametric data (TS, TC, and RPE) were analyzed using R (version 4.0.2). These non-parametric data were analyzed using a two-way [trial (2 levels: FCJ and CON) × time (6 levels: 30, 35, 50, 65, 70, and 75 min)] repeated measures ANOVA with the R package nparLD (version 2.1) (Noguchi et al., 2012). Pair-wise differences between trials were evaluated using the Bonferroni multiple comparison tests. In all other cases, statistical analyses of data were done in the IBM SPSS (version 21; IBM Corp., Armonk, NY, United States). Data collected over time (skin temperatures, T_*ty*_ and HR) were analyzed using a two-way [trial (2 levels: FCJ and CON) × time (6 levels: 30, 35, 50, 65, 70, and 75 min)] repeated measures ANOVA, and single time point data (Pre-exercise variables, the volume of water ingested, body mass loss, total sweat loss and the rate of fall in each variable during both recovery periods) were analyzed using paired sample *t*-tests. Cohen’s d (*d*) was used as a measure of effect size for parametric paired samples; a *d* of 0.2 to <0.5 and ≥0.5 to <0.8 has been indicated to represent a small and medium treatment effect, respectively, while a *d* ≥ 0.8 represents a large treatment effect (Cohen, 1988).

## Results

### Baseline Characteristics

**FIGURE 3:**
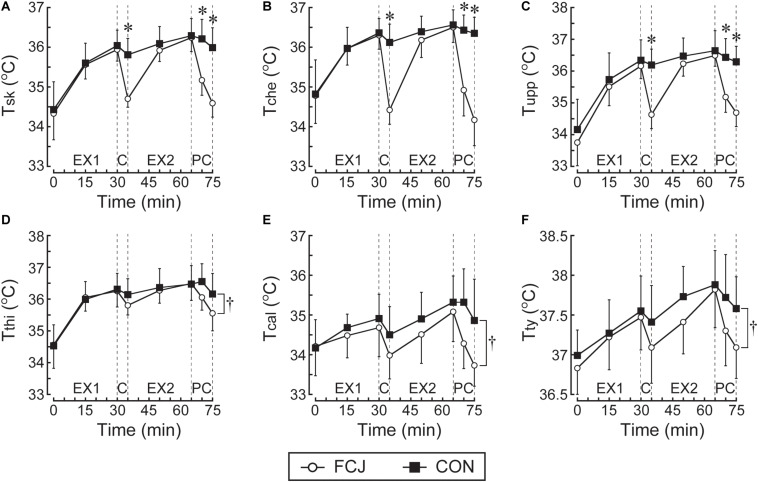
Responses of mean **(A)**, T_*sk*_; chest **(B)**, T_*che*_; upper arm **(C)**, T_*upp*_; thigh **(D)**, T_*thi*_; and calf **(E)**, T_*cal*_; skin temperatures and infrared tympanic temperature **(F)** T_*ty*_. EX1, the first exercise bout; C, the inter-exercise recovery; EX2, the second exercise bout; PC, the post-exercise recovery. *P < 0.001 denotes significant differences between FCJ and CON trials at particular time points. ^†^*P* < 0.05 denotes a significant two-way interaction of trial × time between FCJ and CON trials during the experimental trials.

**FIGURE 4:**
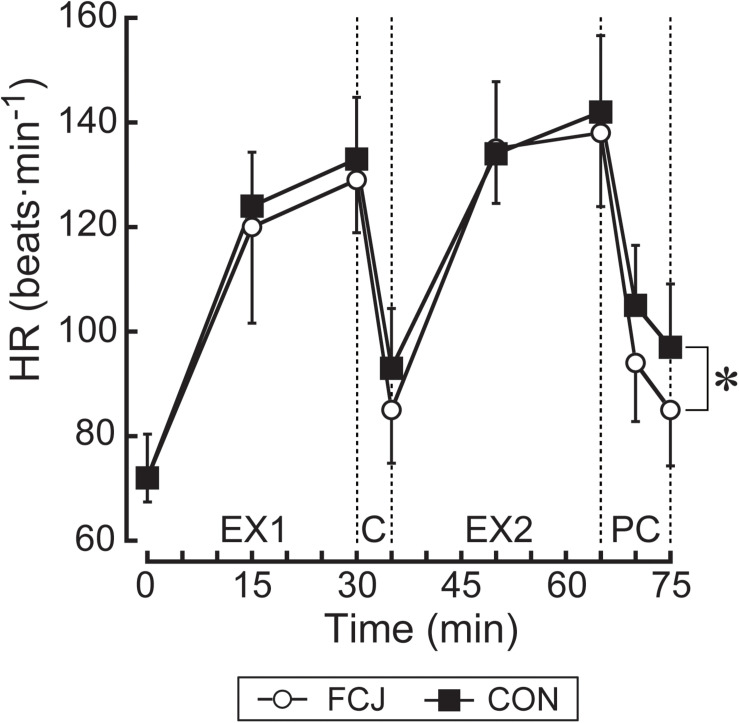
Changes in heart rate (HR). EX1, the first exercise bout; C, the inter-exercise recovery; EX2, the second exercise bout; PC, the post-exercise recovery. *P < 0.05 denotes a significant two-way interaction of trial × time between FCJ and CON trials during the experimental trials.

Pre-exercise body mass (FCJ 66.4 ± 6.9 kg; CON 66.7 ± 6.4 kg: *P* = 0.924), T_*sk*_ (*P* = 0.793), T_*ty*_ (*P* = 0.351), and HR (*P* = 0.970) were not different between trials. During the first exercise bout, there were no differences between trials in all variables (all *P* > 0.05; Figures 3, 4).

### Skin Temperature Responses

T_*sk*_, T_*che*_, and T_*upp*_ were lower on FCJ than CON trial at 35, 70, and 75 min (all *P* < 0.001; Figures 3A-C). T_*thi*_ and T_*cal*_ were lower during the experimental trials on FCJ than CON trial (both *P* < 0.05; Figures 3D,E).

The rates of fall in T_*sk*_, T_*che*_, and T_*upp*_ were faster during both inter-exercise and post-exercise recovery on FCJ than CON trial (Table 1). There were faster rates of fall in T_*thi*_ and T_*cal*_ during the post-exercise recovery on FCJ than CON trial (Table 1).

**TABLE 1:** The rate of fall in each variable during the 5 min inter-exercise (Inter-exer rec) and 10 min post-exercise (Post-exer rec) recovery.

	FCJ trial	CON trial	*P* value	Effect size
**T_*sk*_**				
Inter-exer rec, °C⋅min^–1^	0.2490.054	0.0450.044	< 0.001	4.12
Post-exer rec, °C⋅min^–1^	0.1650.027	0.0300.018	< 0.001	5.96
**T_*che*_**				
Inter-exer rec, °C⋅min^–1^	0.3760.054	0.0470.050	< 0.001	6.32
Post-exer rec, °C⋅min^–1^	0.2330.044	0.0210.031	< 0.001	5.60
**T_*upp*_**				
Inter-exer rec, °C⋅min^–1^	0.3050.078	0.0310.070	< 0.001	3.68
Post-exer rec, °C⋅min^–1^	0.1800.042	0.0360.031	< 0.001	3.93
**T_*thi*_**				
Inter-exer rec, °C⋅min^–1^	0.0900.083	0.0340.072	0.147	0.72
Post-exer rec, °C⋅min^–1^	0.0930.044	0.0310.032	0.001	1.61
**T_*cal*_**				
Inter-exer rec, °C⋅min^–1^	0.1320.088	0.0730.068	0.216	0.75
Post-exer rec, °C⋅min^–1^	0.1120.057	0.0360.052	0.004	1.40
**T_*ty*_**				
Inter-exer rec, °C⋅min^–1^	0.0770.046	0.0280.041	0.042	1.13
Post-exer rec, °C⋅min^–1^	0.0750.026	0.0300.014	0.001	2.07
**HR**				
Inter-exer rec, bpm⋅min^–1^	8.82.1	7.92.1	0.401	0.42
Post-exer rec, bpm⋅min^–1^	5.31.0	4.40.6	0.045	1.04
**TS**				
Inter-exer rec, TS⋅min^–1^	0.80.3	0.20.2	< 0.001	2.50
Post-exer rec, TS⋅min^–1^	0.50.2	0.20.2	0.008	1.36
**TC**				
Inter-exer rec, TC⋅min^–1^	0.40.1	0.20.2	0.007	1.44
Post-exer rec, TC⋅min^–1^	0.20.1	0.10.1	0.104	0.49
**RPE**				
Inter-exer rec, RPE⋅min^–1^	1.10.5	1.00.6	0.591	0.23

*Values are mean ± SD. Effect size is calculated as Cohen’s *d*. T_*sk*_, mean skin temperature; T_*che*_, chest skin temperature; T_*upp*_, upper arm skin temperature; T_*thi*_, thigh skin temperature; T_*cal*_, calf skin temperature; T_*ty*_, infrared tympanic temperature; HR, heart rate; TS, thermal sensation; TC, thermal comfort; RPE, rating of perceived exertion; bpm, beats⋅min^–1^.*

### Infrared Tympanic Temperature Responses

T_*ty*_ was lower during the experimental trials (*P* < 0.001) on FCJ than CON trial (Figure 3F).

There was the faster rate of fall in T_*ty*_ during both inter-exercise and post-exercise recovery on FCJ than CON trial (Table 1).

### Heart Rate Responses

HR was lower during the experimental trials on FCJ than CON trial (*P* < 0.05; Figure 4).

The rate of fall in HR was faster during the post-exercise recovery on FCJ than CON trial (Table 1).

### Body Fluid Balance

No statistical differences were seen in the volume of water ingested (FCJ 1.14 ± 0.48 L; CON 1.23 ± 0.42 L: *P* = 0.676), body mass loss (FCJ −0.1 ± 0.5 kg; CON −0.1 ± 0.4 kg: *P* = 0.892) and total sweat loss (FCJ 1.0 ± 0.2 kg; CON 1.1 ± 0.4 kg: *P* = 0.290).

### Perceptual Responses

**FIGURE 5:**
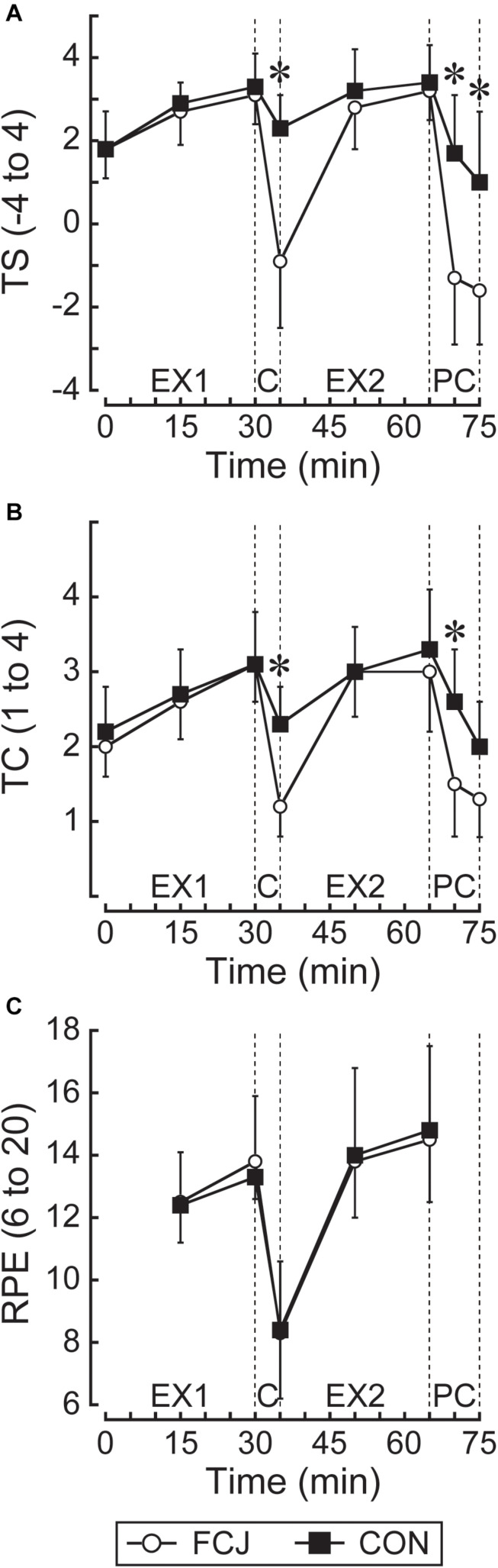
Responses of thermal sensation **(A)** TS (–4 extremely cold to 4 extremely hot); and comfort **(B)** TC (1 comfortable to 4 very uncomfortable); and rating of perceived exertion **(C)** RPE (6 no exertion at all to 20 maximal exertion). EX1, the first exercise bout; C, the inter-exercise recovery; EX2, the second exercise bout; PC, the post-exercise recovery. *P < 0.05 denotes significant differences between FCJ and CON trials at particular time points.

TS was lower on FCJ than CON trial at 35, 70, and 75 min (all *P* < 0.01; Figure 5A). TC was lower on FCJ than CON trial at 35 and 70 min (*P* < 0.01 and *P* < 0.05, respectively; Figure 5B). There was no statistical difference between trials in RPE (*P* = 0.695; Figure 5C).

There was the faster rate of fall in TS during both inter-exercise and post-exercise recovery on FCJ than CON trial (Table 1). The rate of fall in TC was faster during the inter-exercise recovery on FCJ than CON trial (Table 1).

## Discussion

In support of the experimental hypothesis, FCJ trial attenuated increases in thermal (T_*sk*_, T_*che*_, and T_*upp*_) and perceptual (TS and TC) strain during and following exercise in comparison to CON trial (Figures 3, 5). FCJ rather than CON trial also reduced T_*thi*_, T_*cal*_, T_*ty*_ and HR during the experimental trials (Figures 3, 4), albeit there was the absence of RPE difference between trials (Figure 5C). These observations were associated with faster rates of fall in T_*sk*_, T_*che*_, T_*upp*_, T_*ty*_, and TS during both inter- and post-exercise recovery (Table 1). Faster rates of fall in TC during the inter-exercise recovery and in T_*thi*_, T_*cal*_, and HR during the post-exercise recovery were also obtained in FCJ than CON trial (Table 1). A novel finding in the current study is therefore that cooling between exercise bouts and post-exercise with a commercially available fan cooling jacket would effectively reduce thermal strain and perception/discomfort during and following exercise in compensable hot-humid environments.

In the current study, a lower T_*sk*_ during exercise was observed in with than without the fan cooling jacket with skin wetting by sweating (Figure 3A). Nevertheless, T_*ty*_ and HR during the experimental trials were lower in with than without the fan cooling jacket with skin wetting by sweating, but *post hoc* testing revealed no difference in T_*ty*_ and HR between them at any time point during exercise (Figures 3F, 4). Previous studies employing cooling between exercise bouts with an electric fan with skin wetting by externally applied water to the skin surface in uncompensable heat stress (Mitchell et al., 2001; Schranner et al., 2017; Lynch et al., 2018) showed a lower T_*sk*_, T_*core*_, and HR during exercise in with than without a fan trial. Meanwhile, previous studies employing cooling between exercise bouts with upper body cooling garments (e.g., an ice-vest/jacket) in compensable heat stress (Duffield et al., 2003; Cleary et al., 2014; Chaen et al., 2019) have reported a lower T_*sk*_ in with than without garment use trial but the absence of T_*core*_ and HR differences between trials during exercise. Although there is an inter-study difference in T_*core*_ and HR responses, based on previous studies and the current study, cooling between exercise bouts with a fan with skin wetting and with upper body cooling garments would effectively reduce T_*sk*_ during subsequent exercise in the heat.

Although the fan cooling jacket can cool off the upper-body only, T_*sk*_ was lower and the rate of fall in T_*sk*_ was faster during both inter- and post-exercise recovery in FCJ than CON trial (Figure 3 and Table 1). This indicates that the use of this fan cooling jacket would reduce whole-body skin temperature quickly within less than 5 min during recovery following exercise under our experimental settings. FCJ trial also demonstrated the faster rate of fall in upper-body skin temperature (T_*che*_ and T_*upp*_) than CON trial but no differences in the rate of fall in lower-body skin temperature (T_*thi*_ and T_*cal*_) between trials during the inter-exercise recovery (Table 1). This might cause a lower upper-body skin temperature in FCJ than CON trial but the absence of lower-body skin temperature differences between trials during the second exercise bout (Figure 3). During the post-exercise recovery, there were faster rates of fall in lower-body as well as upper-body skin temperatures in FCJ trial in comparison to CON trial (Table 1). These findings are consistent with previous studies that used upper-body cooling with an ice-vest (Kim et al., 2020) or whole-body fanning (Carter et al., 1999). These studies have reported lower-body as well as upper-body skin temperatures during the post-exercise recovery in compensable (30°C) (Kim et al., 2020), or uncompensable (40°C) (Carter et al., 1999) heat stresses. These observations may indicate that the fan cooling jacket use reduces whole-body skin temperature quickly while promoting falls in upper-body skin temperature within less than 5 min and lower-body skin temperature at more than 5 min but less than 10 min during recovery following exercise under our experimental settings.

It is known that whole-body fanning during exercise in compensable heat stress could enhance endurance exercise capacity associated with a lower T_*sk*_ and a greater evaporative heat loss with increasing airflow (Saunders et al., 2005; Otani et al., 2018b). Concomitant with this, convective heat loss was greater when airflow was greater than 2.8 m⋅s^–1^ compared with no airflow trial (Otani et al., 2018b). It is recognized that airflow would contribute to heat loss via convection by increasing the convective heat loss gradient in compensable heat stress (Saunders et al., 2005) as this study. Evaporative heat loss is known to dissipate more heat from the skin with increasing airflow (Havenith et al., 1999) and compared with dry (convection + radiation) heat loss during exercise-heat stress (Gagnon et al., 2013). Although the present study did not calculate these heat losses, a decrease in T_*sk*_ from 36.0 to 34.7°C observed in the inter-exercise recovery in FCJ trial would be the result of greater evaporative and convective heat loss. The same would apply to a decrease in T_*sk*_ during the post-exercise recovery in FCJ trial. Previous studies showed that a decrease in skin temperature by fan cooling during the post-exercise recovery in the heat resulted in a faster fall in HR and lower T_*core*_ compared with no fan cooling (Carter et al., 1999) or cooling by ice packs and intravenous chilled saline infusion (Sinclair et al., 2009). Rowell et al. (1969) clearly showed that a decrease in skin temperature by whole-body cooling with a water perfusion suit during uninterrupted exercise would cause cutaneous venoconstriction, which increases the return of blood to the central circulation. This could result in decreases in HR and T_*core*_ (rectal and right atrial blood temperatures) in parallel with a decrease in cardiac output and increases in stroke volume and aortic pressure (Rowell et al., 1969). These responses with a lower and faster rate of fall in T_*sk*_ may contribute to a faster rate of fall in HR during the post-exercise recovery and T_*ty*_ during both inter- and post-exercise recovery in FCJ than CON trial (Table 1), albeit the study of Rowell et al. (1969) did not assess these responses during recovery from exercise.

In line with many previous studies using cooling between exercise bouts (Duffield et al., 2003; Cleary et al., 2014; Schranner et al., 2017; Lynch et al., 2018; Naito et al., 2018; Chaen et al., 2019; Chalmers et al., 2019) and post-exercise (Kim et al., 2020), lower TS and/or TC were apparent during and following exercise in with (FCJ) than without (CON) cooling trial in this study (Figures 5A,B). This indicates that cooling between exercise bouts and post-exercise with the fan cooling jacket would be an effective method to lower thermal perceptual and discomfort during and following exercise in compensable hot-humid environments. Meanwhile, we observed the absence of RPE difference during exercise between with and without cooling trials (Figure 5C) that is consistent with (Duffield et al., 2003; Cleary et al., 2014; Lynch et al., 2018; Naito et al., 2018), or inconsistent with (Schranner et al., 2017; Chaen et al., 2019; Chalmers et al., 2019) these previous studies employing cooling between exercise bouts. The lack of literature agreement might be attributed to differences in cooling strategy and duration, environmental conditions, the exercise intensity and the participant’s fitness level employed between studies. The increase in RPE has been known to track increases in T_*sk*_ (Castle et al., 2012) and T_*core*_ and HR (Nybo and Nielsen, 2001) during exercise-heat stress. In the present study, there was lower T_*sk*_, T_*ty*_, and HR during the experimental trials in FCJ than CON trial, but T_*sk*_ and HR during the second exercise bout at 50 and 65 min were similar between trials. Therefore, it is likely that the 5-min cooling between exercise bouts with the fan cooling jacket would not have enough time for significant reduced RPE during subsequent exercise in the heat. Since no studies have tested this time effect, further investigations are required.

## Limitations

Although rectal temperature has been well known to be standard methods for T_*core*_ assessment in the laboratory settings, we did not use it because we had only a non-disposable rectal thermistor that use had the risk for COVID-19 infection. Even though this study employed T_*ty*_ with a disposable probe cover to avoid this risk, previous studies reported that rectal temperature relates to Otani et al. (2020) or does not relate to Casa et al. (2007) T_*ty*_ during exercise in the heat. Meanwhile, since the current study was conducted under conditions when skin temperature was higher than ambient temperature, ambient air into the clothing microclimate could dissipate heat from the body through both convective and evaporative heat losses. However, in the condition that ambient temperature exceeds skin temperature, this air would cause convective heat gain to the body from the environment and the body would dissipate heat through sweat evaporation only. Under these conditions, the fan cooling jacket use without skin wetting by sweating might result in the increased convective heat gain to the body; in fact, that observation was reported by the study of Wang et al. (2020) employing the fan cooling jacket and trousers during walking under uncompensable heat stress. Moreover, many Japanese manual workers use this type of fan cooling jacket/vest during work-heat stress, especially in outdoors, since a few years ago. However, the current study was not carried out in a natural outdoor environment under the sun because environmental conditions had to be kept constant. In real outdoors settings in the heat of summer, endurance performance decreases and thermal strain increases with rising solar radiation during exercise (Otani et al., 2019b). Furthermore, a progressive increase in environmental heat stress with rising solar radiation and elevation angle during the morning results in a greater thermal strain during exercise in the heat outdoors under a clear sky, compared with a progressive decrease in environmental heat stress with falling solar radiation and elevation angle during the late afternoon (Otani et al., 2017, 2019a). Therefore, future research should employ rectal temperature and carry out in conditions of higher ambient temperature compared to skin temperature and in the heat outdoors under a clear sky. These researches would provide a greater validity and reliability in the study regarding the effectiveness of the fan cooling jacket use during exercise-/work-heat stress.

## Conclusion

The current study indicates that cooling between exercise bouts and post-exercise with a commercially available fan cooling jacket would effectively mitigate thermal strain and lower thermal perception/discomfort during and following exercise in compensable hot-humid environments. These findings would be accompanied by a reduction in whole-body skin temperature quickly while promoting falls in upper-body skin temperature within less than 5 min and lower-body skin temperature at more than 5 min but less than 10 min during recovery following exercise in the heat. These observations suggest that the use of the fan cooling jacket with skin wetting at inter- and post-exercise/work would provide effective cooling for sports participants and workers to reduce the risks of thermal strain and exertional heat-related illness in hot-humid conditions.

## Data Availability Statement

The raw data supporting the conclusions of this article will be made available by the authors, without undue reservation.

## Ethics Statement

The studies involving human participants were reviewed and approved by The ASICS Ethical Advisory Committee. The patients/participants provided their written informed consent to participate in this study.

## Author Contributions

HO and MF conceived and designed the research, performed the experiments, and analyzed the data. HO drafted, edited, and revised the manuscript. All authors interpreted results of experiments and approved final version of the manuscript.

## Conflict of Interest

The authors declare that the research was conducted in the absence of any commercial or financial relationships that could be construed as a potential conflict of interest.
